# Supplementary material for: Association of Primary Sjögren’s Syndrome and Vitamin B12 Deficiency: A Cross-Sectional Case-Control Study
Source: J Clin Med. 2020 Dec 16;9(12):4063. doi: 10.3390/jcm9124063 (PMC7765802; doi:10.3390/jcm9124063)
Supplement: Supplementary file 1 [file jcm-09-04063-s001.pdf]

**Table S1.** Characteristics of pSS patients according to B12 status.

| | **B12 deficiency** | **No B12 deficiency** |
|---|---|---|
| Number of patients | 9 | 12 |
| General characteristics | | |
| Age (years) | 57 [49-77] | 71.5 [60.8-75] |
| Body mass index (kg/m²) | 24.6 [22.2-25.5] | 25.9 [22.5-32.7] |
| pSS duration at time of B12 measurement (months) | 13 [5-46] | 9 [0-42.5] |
| Biological measurements | | |
| Plasma vitamin B12 (ng/L) | 293 [252-304] | 504 [440.3-573] |
| Serum folates (µg/L) | 4.7 [3.8-4.9] | 5.9 [5.1-10.3] |
| Serum ferritin level (µg/L) | 57 [39-115] | 126 [72.8-184] |
| Folates deficiency | 3 (33.3%) | 0 |
| Iron deficiency | 4 (44.4%) | 2 (16.7%) |
| Creatinine clearance (mL/min/1.73m²) MDRD | 75.7 [66.1-92.0] | 94.4 [79.3-101.7] |
| Hemoglobin (g/L) | 121 [113-137] | 135 [129-142] |
| Mean red globule volume (fL) | 89.4 [86.4-93.4] | 91.4 [90.0-93.6] |
| Platelets (G/L) | 254 [216-299] | 241 [218-279] |
| Leucocytes (G/L) | 7.4 [4.8-8.0] | 5.9 [4.9-6.1] |
| Other causes of B12 deficiency | | |
| Metformine use | 0 | 1 (8.3%) |
| Chronic antacid use | 5 (55.6%) | 1 (8.3%) |
| Nutritional deficiency | 0 | 0 |
| Fundic gastric or ileum resection | 1 (11.1%) | 0 |
| *Helicobacter pylori* gastric infection | 0 | 0 |
| Characteristics of pSS | | |
| Age at pSS diagnosis (years) | 56 [48-66] | 64.5 [51.5-71] |
| pSS ACR/EULAR Criteria | | |
| Schirmer's test ≤ 5mm/5min | 5 (55.6%) | 8 (66.7%) |
| Unstimulated whole saliva flow ≤ 1,5ml/15min | 4 (44.4%) | 8 (66.7%) |
| Anti-SSA antibodies | 2 (22.2%) | 5 (41.7%) |
| Lymphocytic sialadenitis with focus score ≥ 1 | 9 (100%) | 11 (91.7%) |
| Extra-glandular manifestations | 6 (66.7%) | 9 (75%) |
| Inflammatory arthralgia | 2 (22.2%) | 5 (41.7%) |
| Polyarthritis | 0 | 1 (8.3%) |
| Parotidomegaly | 3 (33.3%) | 1 (8.3%) |
| Seritis | 1 (11.1%) | 0 |
| Large fiber neuropathy | 1 (11.1%) | 2 (16.7%) |
| Small fiber neuropathy | 1 (11.1%) | 1 (8.3%) |
| CNS involvement | 1 (11.1%) | 1 (8.3%) |
| Pulmonary interstitial disease | 0 | 2 (16.7%) |
| Lymphoma | 0 | 1 (8.3%) |

Notes: CNS: Central nervous system; MDRD: modification of diet in renal disease; pSS: primary Sjögren's syndrome.
